# Practical Observations on the Treatment of the Scarlet Fever and Sore Throat

**Published:** 1804-07-01

**Authors:** 


### Practical Observations on the Treatment of the Scarlet Fever and Sore Throat.

HAVING lately read several publications on the scarlet fever and sore throat, in which are recommended remedies agreeable to the prevailing theories and speculative opinions of the day; I beg leave to lay before your readers a practice in that disease I have, for upwards of thirty years, invariably followed with eminent success.

It is well known that many pass very safely through the scarlet fever in its mild state with little or no medical assistance. But when in that state, medicines are administered, I fear the cure is, by the ingenious theoretical practitioner, ascribed too often to their effects and not to the mildness of the disease; especially if some fashionable medicine has been prescribed; hence remedies undeservedly creep into practice, and, I fear, in serious cases, frequently supercede the use of those which have long stood the test of sound practical experience.

I pretend not to account for the source or origin of the scarlet fever and sore throat, but am well satisfied that the fomes morbi of the disease, however generated, lurk in the bowels. Under this conviction, I strictly enjoin them to be be well cleared in whatever stage or however violent the disease may be, when I first see the patient, if I suspect that such necessary treatment has not been before observed. The very fœtid smell of the evacuation, and the relief such evacuation invariably procures, strongly prove to me the necessity of purgatives; and I may add, from reiterated observations, that the longer they are delayed the more severe proves the disease. Many practitioners, alarmed at apparent debility, are deterred from exhibiting brisk cathartics, lest their operation should irrecoverably sink the patient. Such apprehensions would be justly founded if purgatives were administered without due discriminating attention to the age, constitution, and immediate state of the patient. But where such attention is paid, I have never seen any mischief to arise; on the contrary, the most salutary effects have taken place, merely from the bowels being relieved from the contained accumulated fœtid fæces, and hence every febrile symptom becomes milder, and the vital powers invigorated not debilitated. In the commencement of the disease, I generally prescribe preparations of senna, with or without, as circumstances indicate, the magnesia vitriol.; to these I add, in robust constitutions or even in weakly habits, if pains in the head or sickness in the stomach be urgent, a due proportion of antim. tart. By these means I effect a thorough clearance of the stomach and intestines; when that is accomplished I endeavour to bring on a moderate perspiration by a liberal use of the aq. amm. acet. to which I sometimes add the vin. antim. and sometimes the conf. damoc. I generally direct the feet to be immersed in warm water with one-eighth part of vinegar. This pediluvium I find to be a pleasant and effectual assistant in promoting perspiration and inducing sleep. A pursuance of this plan, interposing at intervals cathartics as occasion may require, will in a few days carry off the first febrile symptoms. The remaining debility becomes then the object of attention. To remove this, light preparations of bark, with a suitable diet, I find most effectual remedies.

The early exhibition of the bark, wine, &c. &c. in the scarlet fever and sore throat, I am persuaded has been, and continues to be, productive of very serious mischief. To such practice, I partly ascribe in many instances the severity, if not the mortality of the disease. Lassitude, prostration of stength, general pains in the limbs, quick pulse, parched skin with an appearing efflorescence, soreness of throat, with enlarged and ulcerated tonsils, denote the comcommenced disease. The practitioner, dreading the rapid progress of the angina maligna in debility and fatal putrefaction, immediately begins to prevent the devastation by plentifully throwing in bark, wine, and other cordials, preparing (or perhaps not) the bowels for their reception by a mild aperient or gentle enema; hence, the very remedies administered with the idea of invigorating the system to resist the much dreaded debility and putrefactive process, become, by retaining the putrid colluvies in the bowels, and irritating the general system, active agents in promoting the very evils they were intended to avert.

The vitriolic acid diluted with aq. dis. to which I add a sufficient quantity of syr. papav. errat. forms a gargle pleasant to the eye and taste. With this gargle I strenuously recommend the tonsils, fauces, and mouth to be kept clean, and that it should be used always immediately before nourishment is taken down.

I very rarely make use of any other external application, being long doubtful whether blisters, by the pain and trouble they occasion, compensate for their *supposed* good effect.

It often happens, that in children the gargle cannot be used, and the sloughs and foul mucus will accumulate to a very alarming degree. In such cases I have relieved the little patients from the stupor and suffocation, under which they appeared dying, by emetics, and have, by their timely use in some children, prevented the accumulation from taking place.

*May* 21, 1804. MEDICUS.

